# Supplementary material for: Are carbon emissions trading and green financial instruments synergistic? -Comprehensive quantitative research based on content analysis
Source: PLoS One. 2024 Mar 7;19(3):e0298601. doi: 10.1371/journal.pone.0298601 (PMC10919601; doi:10.1371/journal.pone.0298601)
Supplement: S1 Data — (ZIP) [file pone.0298601.s001.zip › Data/地级市Z_F工作报告.zip!/╜¡╦╒/╦▐╟¿╩╨/└┤╫╘╣1⁄2╓┌║┼í╛┬φ┐╦╩2╛▌═°í┐_2015.docx]

各位代表：

现在，我代表宿迁市人民政府，向大会作工作报告，请予审议，并请各位政协委员和其他列席同志提出意见。

2014年工作回顾

过去的一年，在省委、省政府和市委的坚强领导下，在市人大、市政协的监督支持下，市政府深入贯彻落实党的十八大、十八届三中四中全会和习近平总书记系列重要讲话精神，围绕全面建设小康社会的奋斗目标，团结和依靠全市人民，统筹做好稳增长、抓改革、调结构、惠民生等各项工作，经济社会发展呈现稳中快进的好势头。预计地区生产总值突破1800亿元、达1860亿元，同比增长10.5%；公共财政预算收入突破200亿元、达210.1亿元，增长13.5%；固定资产投资突破1500亿元、达1550亿元，增长20.1%；全体居民人均可支配收入突破15000元、达15929元，增长11.3%。虽然发展速度告别了突飞猛进的增长阶段，但主要指标增幅依然领跑全省，在省列统的16项主要经济指标中，有7项位居全省前列、5项位居第一。

一是经济运行健康有序。面对复杂多变的宏观形势，牢牢把握“稳”与“进”的平衡点、结合点，经济运行呈现结构持续优化、质量稳步提升的良好态势。工业经济量质齐升。规上工业总产值和销售收入双双超过3000亿元，工业固定资产投资超过1000亿元、相当于1996年至2009年14年的累计投入，工业经济总体实力迈上新台阶；新增销售超10亿元以上企业11户，一年新增数量是以往年均增量的5倍，企业实力稳步增强；新增场外市场挂牌企业18户，“新三板”挂牌数位居苏北首位；重工业产值首次超越轻工业，工业用电量突破100亿千瓦时，但单位GDP能耗五年来首次下降，新兴产业和高新技术产业产值占比分别提高到23.3%、19.9%，工业结构进一步调高、调新、调优；第一台冰箱、制冷压缩机和第一部手机成功下线，改变了长期以食品、板材等为主的产品结构。招商引资成果喜人。康师傅饮品、杉杉服饰、海天酱油等一批知名企业纷纷落户，新开工5亿元以上大项目创历史新高；实际利用外资增长34%，新批及净增资3000万美元以上大项目25个、是2013年的2.5倍，外资招引逆势飘红。园区发展进位争先。市及三县两区开发区省内排名全面进位；5个共建园区进入全省共建园区前10名，苏宿园区连续五年排名第一。服务业发展步伐加快。增加值增速连续两年高于地区生产总值，入库地方税收突破百亿；旅游业收入达145.7亿元，增长44.7%、居全省第一；南京银行、长江商业银行相继落户，率先实现省内四大城市商业银行全覆盖；华东云计算中心等项目顺利推进，中国宿迁电子商务产业园正式揭牌，全省首家电子商务秘书公司成立运营，电子商务产业蓬勃兴起。现代农业基础不断夯实。粮食生产“十一连丰”，小麦单产、总产再创历史新高；2/3以上知名国字号农业龙头企业在宿建设加工基地，家庭农场总量和认定“三品”产地面积占比均位居全省首位；规模养殖比重位居全省前列，河蟹出口量连续九年全省第一。

二是主导产业加速集聚。出台促进产业集聚发展意见，构建了市域“双新一特”和县域“2+1”产业体系，产业导向更加聚焦。“三电一绿”产业迅速崛起，智能家电、绿色建材产业仅用一年多时间就形成了集聚态势，双双获批为省级先进制造业基地。家电产业平地崛起。集聚了格力电器、东贝压缩机、双鹿上菱电器等总投资超百亿元的13个大项目，形成了较为完备的产业链条，初步确立了全国白色家电产业第五极的地位。光电产业高端成长。吉创光电研制技术领先全国的5.5代投射式电容触摸屏，已进入试生产阶段，宿迁有望成为全国顶级电容触摸屏生产基地。机电装备产业迅猛发展。可成科技、长电科技等一批龙头企业产能充分释放，机电装备产业仅用四年即实现产值从百亿级到500亿级的跨越；可成科技投产四年产值增长50倍、突破60亿元，今年有望成为继苏酒集团之后全市第二家百亿级企业。绿色建材产业集聚成势。连续两年主办绿色建材产业合作论坛，在德国成功举行绿色建材产业专场推介会，与世界500强企业巴斯夫集团签订战略合作协议，德国BGW、康程新材料等总投资超过140亿元的20余个大项目相继落户，绿色建材产业逐渐成为全国有影响的特色产业。县区主导产业特色彰显。沭阳县的装备制造、泗阳县的汽车配件、泗洪县的新型膜材料、宿豫区的新型材料、宿城区的智能电网等产业集聚度不断提升、特色更加鲜明。

三是改革创新率先突破。坚持以市场化为导向，以减权去利为核心，大力度深层次推进各项改革。快人一步率先改。比全国提前近一年开展先照后证、并联审批和“四证（照）一体”改革，大大激发了广大群众创业热情，全年新增公司制企业9801户、较2013年增长78.8%；率先开展网络经营主体工商登记，全国首张个体网店数字营业执照在宿核发；在全国首推资格资质去行政化改革，569项资格资质项目仅保留53项、放开率达81.2%，市场主体参与率超过60%；大力开展资源要素价格改革，签订了全国首例水气热交易合同；在全省率先开展乡镇村级土地股份合作社、农村土地承包经营权抵押贷款和涉农贷款保证保险办理试点改革。减权去利彻底改。行政审批事项由193项压减至57项，精简率超过70%，三县两区均压减到50项以内，成为全国行政审批事项最少的地区之一；工商登记前置审批事项由199项压减为7项，精简率超过96%，企业注册登记基本不需前置审批，办理营业执照由原来至少1个月压减到5天左右；中介机构与行政机关脱钩，斩断了权力寻租的利益输送链条。层层递进有序改。以商事登记制度改革为突破口，开展行政审批制度、资格资质去行政化、非行政许可审批事项清理“三部曲”改革，着力管住政府“闲不住的手”；围绕“使市场在资源配置中起决定性作用”，推进资源要素价格、政府购买服务、国资平台等改革，打破垄断和分割，着力放活市场“无形之手”；着眼构建现代化社会治理模式，开展社会组织培育和信用体系建设，着力培育社会“自治之手”。环环相扣系统改。围绕“放”，最大限度压减审批事项、减少审批环节，激发了市场活力；围绕“退”，全面推进政企分离、政社分离，实现了社会组织与政府部门脱钩；围绕“接”，降低社会组织准入门槛，新增社会组织2202个，相当于历史总和；围绕“管”，建立监管联动平台和社会信用体系，并逐项制定所有审批事项的事中事后监管办法，实现了宽进严管。这一系列改革举措，既充分激发出市场和社会主体活力，也高度契合中央改革意图，准确把握了改革核心要义，特别是资格资质去行政化改革受到中央领导肯定和批示，央视新闻联播两次报道我市改革。

四是城市形象崭露新姿。围绕“最美的生态、永远的时尚”建设定位，完善功能配套，塑造城市个性，增强了市民的归属感、幸福感和自豪感。重大项目取得突破性进展。通用机场项目完成初步选址，宿豫陆运河项目正式获批，中心城市迎来首条宿淮铁路客货专线；徐宿淮盐高铁项目通过工可审查，将于上半年破土动工，宿迁人民期盼已久的“高铁梦”将要成为现实。城市功能不断完善。以运河宿迁港为代表的中心城市180项重点工程顺利推进，项王故里景区二期建成运营，运河南路、八一西路改造等工程全面完成，325省道淮安至宿迁段、344省道沭阳段建成通车，新（改）建“两场一街一中心”项目30个，城市服务功能进一步提升。城市个性日益凸显。全面完成151项园林绿化项目，新植各类乔灌木17.3万株，新增绿地810万平方米，建成一批彩色林荫大道、花海花廊和落叶景观道，运河桥头公园、环城西路景观绿化等公园游园建成开放，古黄河雄壮河湾南岸景观工程荣获国内风景园林领域最高奖，城市环境更加清新怡人；三台山扩面提质工程全面启动，致力打造“最美”系列，赋予城市新的特色和亮点。城市文明加快培育。丰富“宿迁规矩”内涵，扎实推进交通秩序整治，礼让斑马线行为全省最好，交通文明指数全省第一；全面清除各类非法小广告，楼道内、墙壁上的“牛皮癣”明显减少；首批54个小区物业整治顺利推进，市区2500多家小餐饮整治全面完成，成功做法在全省推广；常态开展志愿服务、文明宣导等活动，涌现出一批先进集体和模范人物，文明有礼成为宿迁人的新特质。“五城同创”实现整体突破。国家卫生城市已正式公示，节水型城市通过国家现场考核，环保模范城市通过省级评估、即将迎来国家评估，人居环境奖城市通过省级考核验收，文明城市通过省级暗访测评、直接晋升全国提名城市，持续不断的创建活动既完善了城市功能、优化了环境，也改善了民生、凝聚了民心。县城特色不断彰显。沭阳县国际图书城、泗阳县妈祖文化园、泗洪县世纪公园改造等一批标志性项目建成启用，沭阳县、泗洪县创成省卫生县城，泗阳县通过国家园林县城省级评审，获评“中国最美丽县城”称号。

五是民生改善紧扣需求。公共财政支出近七成用于民生，民生幸福“六大体系”发展指数跃居苏北首位。民生实事项目稳步实施。民生六项工程扎实推进，76项为民办实事项目顺利完工；全面完成15个老旧小区、10个老旧农贸市场改造任务，惠及城区近十分之一人口；改造66个老小区燃气设施，低保户、特困户享受安装费用减免；城区公共厕所全部达到三类以上水冲式公厕标准，且全部免费开放；新开工各类保障性住房4.2万套、基本建成3.6万套，圆了更多困难群众的安居梦；完成239家学校食堂整规改造，广大师生“舌尖上的安全”更有保障；93个站点、2350辆公共自行车投放系统建成运行，骑乘公共自行车已成为广大市民交通出行的新方式。创业就业成效明显。城镇新增就业4.8万人，转移农村劳动力3.5万人，“零就业”、“零转移”贫困家庭保持动态清零，城镇登记失业率创历史新低；网络创业成为居民增收新引擎，新增网店1.2万个、正常经营网店达3.1万个，新增全国“淘宝村”2个、总数达4个。社会保障扩面提标。“五险”扩面31.4万人次，城乡低保标准提高到每人每月370元和305元，农村五保集中、分散供养标准提高到每人每年5800元和4600元，城市“三无”老人供养标准提高到每人每年9000元；企业职工基本养老保险覆盖率达95.4%，城镇基本医疗保险和新农合覆盖率均达98%以上，城乡居民社会养老保险覆盖率达98%以上，不断完善的社保体系为广大群众织就了一张“安全网”。“一水一房”建设扎实推进。水源地保护工程启动实施，区域供水和农村饮水安全工程加快推进，又有87万农村群众安全饮水得到保障；新建、加固中小学校舍62万平方米，全市所有中小学D级校舍全部拆除到位。生态环境持续优化。宿迁生态化工科技产业园整治初见成效，马陵河水环境综合整治全面展开，成功入选全国水生态文明城市建设试点市；深入实施大气质量提升专项整治行动，PM2.5、PM10浓度分别下降8.78%和8.13%，空气质量优良率进一步提升。扶贫开发完成阶段性任务。泗洪西南岗、成子湖周边和黄墩湖滞洪区扶贫开发扎实推进，淮沭河沿线扶贫开发拉开序幕，成子湖周边地区上升为省级扶贫开发试验区，我市被列为国家扶贫改革试验区；农村低收入人口脱贫、经济薄弱村新“八有”年度目标顺利实现。

六是社会发展协调并进。教育战线喜讯频传。上一轮中心城市教育布局调整顺利完成；马陵中学、市实小喜迁新址；省优质园占比突破六成；高考本科达线人数连续五年超万人，清华北大录取人数位居全省第四、蝉联苏北第一；宿迁学院成功申本，宿迁人民终于有了自己的独立本科院校。医疗卫生事业稳步发展。社区卫生服务站启动实施基本药物制度，村卫生室实施基本药物制度进一步规范，990个村卫生室完成标准化改造，65岁以上老人实行免费体检，市残疾人康复、托养中心建成并试运行，群众身体健康有了更多保障；市公共卫生服务中心启动建设，市第一人民医院完成外装，市人民医院外科大楼投入使用，市妇产医院、洋河新区人民医院实现整体搬迁，宿迁荣获“健康中国梦—全国民营医院发展示范城市”称号。文化事业亮点纷呈。市博物新馆、图书新馆、档案馆建成投用，文化共享工程服务点实现村级全覆盖，有线电视入户率提高了10个百分点；成功举办首届宿迁市书画家晋省展，承办省美协新人美术作品展；新增中国书协会员10人，中国美协会员从3人增加到7人；入展兰亭奖、获得全国美展提名奖等国内书画重要奖项人次创历史新高；6部作品获省第九届精神文明建设“五个一工程”奖，长篇琴书《白绫记》荣获中国曲艺界最高奖—牡丹奖，大型现代淮海戏《乡村好人》列为省级舞台精品工程；“速新闻”正式上线，龙王庙行宫和中运河段入选大运河世界文化遗产，项王故里新年祈福开门迎宾大典“一炮打响”，一个个“文化事件”浓墨重彩地描绘了宿迁文化事业繁荣发展的美好画卷。科技人才工作实现重大突破。国家高新技术企业突破百家，新增省级高新技术产品突破百个，争取省级以上科技项目实现历史性突破；引进国家“千人计划”专家18人，入选省“双创人才”17人，省双创团队实现“零突破”。体育影响力明显提升。十八届省运会金牌数、奖牌数和总分数全部实现翻番，排名创历史新高；宿迁籍残疾人运动员在仁川亚残会上勇夺“九金一银”，金牌数占全省近1/3；成功举办国际A级赛事—中国•宿迁骆马湖生态四项精英赛，打响了宿迁生态体育品牌。对外交流成果丰硕。省市共建驻德国经贸代表处挂牌运作，与友城新西兰因弗卡吉尔市合作设立国际奖学金，开启了宿迁学子出国留学的“直通车”。电子政务工作走在全国前列。成功举办2014“中国政务微博路在何方”高层论坛，“网上宿迁”跻身中国政府网站绩效评估前三强。社会治安持续向好。平安创建、综合治理、信访稳定等工作扎实推进，安全生产工作连续九年荣获全省目标考核一等奖，群众安全感和满意度位居全省前列。政府自身建设不断加强。惩防体系建设稳步推进，廉政风险防控机制加快完善，党的群众路线教育实践活动取得显著成效，“四风”问题得到明显遏制，政风行风进一步好转。各项事业蓬勃发展。民防、物价、气象、地震、食品药品安全、宗教、计生、人民武装和双拥、档案、党史、地方志等工作实现了新进展，妇女、儿童、青少年、老龄、红十字、慈善等事业取得了新进步。

市四届人大三次会议以来，在宏观形势复杂严峻、面临困难不断增多的情况下，我们立足于谋定后动，精准施策，巩固了科学发展的好势头、政通人和的好局面。回顾去年以来工作，市政府在组织经济工作和社会管理中主要有四个方面鲜明特征：

一是一抓到底，紧盯不放韧性足。说了干、干则成。始终以高度的责任心对待工作，对于认定认准的事情、百姓拥护的事情、承诺兑现的事情，无论问题多难、矛盾多大、标准多高，都能持之以恒、不畏艰难、心无旁骛地抓推进、抓落实，善始善终、善做善成。直面困难不退缩。面对创卫和创建文明城市底子薄、基础差的现状，坚持从基础最薄弱的环节抓起，从公众最需要的地方入手，通过老旧小区改造、公厕、农贸市场、停车位、图书馆、文化馆等配套设施建设，一草一瓦地改善城市环境，一点一滴地提升文明素养，最终把不可能变为可能，把不看好变成叫好，创卫以高分一次性通过国家评估验收。步步深入不松劲。从一个个具体环节入手，从一个个关键步骤发力，环环相扣抓好落实、稳扎稳打解决问题，不达目的不罢休。以整治闯红灯和开展礼让斑马线活动为突破口，持续加大交通秩序整顿力度，通过不断强化的宣传教育和严管重罚，推动“宿迁规矩”内化为“宿迁习惯”，促进了社会良好秩序的建立和巩固。

二是注重细节，制定导则立标准。注重谋划全局，但不大而化之。坚持把标准化、精细化贯穿于工作始终，从小事着眼、细节着手、点滴着力，对每一个项目建设、每一个工程推进，均制定过细的导则和标准，搌细节以成就完美、拢微末以铸就精品。依据导则抓建设。在园林绿化中专门编制建设、管理、养护等一系列导则，不仅明确了栽植范围、形态搭配，连树坑大小、换土质量、晾晒时间、培土高低等都作了详细规定，把绿化施工流程和规范精确到每一个细枝末节。突出细节抓管理。建立城市管理标准导则体系，从工程渣土清运、公厕管理等11个方面制定了规范，尤其是对农贸市场改造，不仅注重在店牌店面等“面子”工程上定规范，更注重在地下管网等“里子”工程上立标准，通过一个个细节的处理和完善，城市管理水平大幅提升。着眼点滴抓整治。专门制定了小餐饮整治“五化”要求，按照不同业态，从硬件设施、加工制作、管理制度，对小餐饮操作区设置、功能区改造、硬件配备、物品摆放等制定了详尽导则，确保了整治成效。

三是尊重市场，锐意改革求突破。坚持市场导向，遵守市场规则，创新发展路径，革除发展障碍。用市场化手段组织经济工作。减少并努力避免政府对微观经济干预，用市场的观念和方法加强要素保障，无论是以银企对接帮助企业破解融资难、以地产品对接帮助企业化解销售难，还是以举办水气热供用交易会帮助企业降低生产成本，均是通过搭建平台、信息对接等方式提供服务，为企业发展创造了一个平权环境。用改革理念化解难题。面对经济发展新常态，开拓思路、创新求变，坚持以改革思维探索破解难题的新路径。在产业发展上，聚焦“双新一特”产业；在招商引资上，由全民招商转向“三位一体”招商；在基础设施建设上，创新采用PPP模式引导社会资本投入；在建筑施工领域，探索开展设计施工一体化改革；在改革实践中，采取赎买收权、暂停行权、争取授权等方式，确保了各项改革有序、有效推进。用创新成效赢得先机。通过实施以“减权、去利、市场化”为导向的一系列改革，使体制更加健全、机制更加灵活、制度更加优越，宿迁改革高地的优势再次彰显，赢得了投资者的青睐，抢占了发展先机，推动经济社会在复杂多变形势下实现了快速健康发展。

四是循法以动，尊重民意聚合力。坚持把民主和法治作为理念追求和行为习惯。依法行政用好权。按照“法无授权不可为”的原则，将行政行为纳入法治框架内考量，施政方式、治理方式更加规范有序、公开透明。专门修订常务会议议事规则，将重大问题决策、重要编制人事、重大项目投资、大额资金使用等纳入审议，避免了个人决策和临时决策；健全重大行政决策程序，在制订2015年部门重要事项过程中，由法制、监察、财政等部门开展合法性、廉洁性及资金来源专项审查；对骆马湖生态治理、扬尘整治等重大问题，提请市人大、市政协专题调研，以决策程序的公开保证了结果的公平公正。政民互动聚民心。创办“您知道吗”栏目，对重大决策、重要政策进行深度解读，让公众第一时间了解掌握有关政策和信息；依托社会论坛建立“鼎鼎有民”网络平台，注册会员发帖量是2013年同期的2倍，月均点击达353万次；开展“全媒体政民互动”，搭建起市领导与广大市民之间的“直通车”，成功举办11期，群众反响十分热烈；在老旧小区改造过程中，坚持改造前倾听群众呼声，改造中强化群众评议，改造后接受群众评判，有效提升了市民的满意度。

各位代表，刚刚过去的2014年，我们在经济社会发展上取得了累累硕果，发展中的宿迁让投资者称赞、老百姓自豪、外地人向往。这一年，创业门槛更低了、手续更简了，越来越成熟的营商环境令投资者点“赞”、创业者心仪；这一年，就业岗位更多了、待遇更好了，越来越多的打工者不再外出，照顾家庭和挣钱两不误；这一年，城市变得更美了、更有序了，老百姓的认可度越来越高、自豪感越来越强；这一年，宿迁汇聚了更多活力，散发出更大魅力，让外地人来了不想走、走了还想来。

这些良好业绩的取得，离不开省委、省政府和市委的正确领导，离不开省级机关和苏州等兄弟市的关心帮助，离不开市人大、市政协和社会各界的监督支持，离不开全市人民的团结一心、奋力拼搏。在此，我代表市人民政府，向奋战在各行各业的全体劳动者，向人大代表、政协委员、各民主党派、工商联和无党派人士、各人民团体，向离退休老同志，向中央和省驻宿单位以及人民解放军、武警官兵、公安干警，向参与宿迁建设的海内外客商、高层次人才、广大援宿干部，以及所有关心支持宿迁发展的各级领导、各界人士，致以崇高的敬意和衷心的感谢！

在肯定成绩的同时，我们也清醒地看到，全市经济社会发展中还存在一些矛盾和问题：经济下行压力加大，部分指标增速回落；实体经济困难加重，部分行业持续低迷；产业质态依然不高，转型升级任重道远；资源要素制约凸显，节能减排压力增大；城乡统筹任务艰巨，公共服务体系不够完善，民生工作尚有不少差距；政府职能有待转变，新常态下组织经济工作方式亟需改进，等等。对此，我们一定高度重视，采取有效措施，认真加以解决。

2015年工作安排

今年政府工作的总体要求是：全面贯彻党的十八大、十八届三中四中全会和省委十二届八次九次全会，以及习总书记系列讲话特别是视察江苏时的重要讲话精神，按照市委四届五次全体（扩大）会议部署安排，围绕“迈上新台阶、建设新宿迁”的发展定位，突出“三抓三促”工作主线，坚持稳中求进，深化改革创新，主动适应和引领经济新常态，积极培育和壮大发展新动能，不断增强区域综合实力和核心竞争力，努力推动经济社会转型发展、绿色发展、创新发展，谱写全面小康社会建设新篇章。

经济社会发展的主要预期目标是：地区生产总值增长10%左右，一般公共预算收入增长11%左右，固定资产投资增长19%左右，社会消费品零售总额增长12.5%左右，居民消费价格涨幅3.5%以内，全体居民人均可支配收入增长10.5%左右，节能减排完成年度目标任务。确定这样的目标，是市委、市政府全面把握宏观经济新常态，审慎考虑宿迁发展实际，权衡各方因素决定的，既遵循了我市经济中长期发展规律，又反映了现阶段经济增长潜力。完成这一目标，保持经济平稳发展，需要充分挖掘发展新动能，加快培育新的经济增长点。重点挖掘七个方面的新动能：

一、以转型升级为关键，增添经济持续健康发展新动能

坚持做大规模与优化结构互动并进，不断提升产业发展的层次、质量和效益，加快构建结构优化、附加值高、竞争力强的现代产业体系，实现更有质量、更高效益、更可持续的发展。

推进工业主导产业集聚升级。以总投资1000亿元的100个新型工业化项目为重点，加快外引内培，强化集聚升级，打造宿迁工业经济“升级版”。推动主导产业集聚。立足全市“4+4”新特产业、县区“2+1”产业发展定位，深入实施主导产业培育三年计划，加快编制产业发展规划，推动主导产业向高端化、集聚化、特色化迈进。因地制宜培育一批产业链或产业树，推动产业垂直整合、横向合作、融合发展；锁定产业体系中的关键环节加以招引和培育，依托宿迁生态化工科技产业园建设印染服务中心和金属表面处理中心，既满足纺织服装、家电等产业发展需求，又实现生产流程“去污化”、“清洁化”。转变招商引资方式。强化“三位一体”招商，提高招商引资的专业化、科学化水平；精心筹办第五届宿迁经贸洽谈会，支持行业组织以及市内外龙头企业在宿举办产品洽谈会、展销会，办好智能家电、绿色建材两场博览会；积极创建中德（宿迁）绿色建材产业发展示范区，力争对德绿色建材产业招商实现更大突破；大力吸引台湾成长型、创新型、科技型项目在宿集聚，争创海峡两岸（宿迁）创新型中小企业发展示范区。支持企业做精做强。以开展“三服务三促进”活动为抓手，组织实施“四大助企行动”，采取市场化的理念和手段，支持实体经济平稳健康运行；以实施企业培育“511”工程为契机，支持企业绿色发展、创新技改、兼并重组、品牌创建、市场开拓，加快培育一批主业强、竞争力强、带动力强的行业领军企业。增强园区支撑功能。按照“五有三转”的要求，推动开发区产业集聚、创新发展，打造永不转移的产业基地；加快综保区申创工作；提升七大经济园的建设水平，积极争创省级工业园区。

加快农业现代化建设步伐。遵循现代农业发展方向，加快转变农业生产经营方式。培育新型经营主体。加大对专业大户、家庭农场的指导和服务力度，推进4个生态家庭农场集群建设，提高经营管理水平和市场竞争力；实施合作社发展质量、服务能力提升工程，支持龙头企业组建大型企业集团，加快培育一支高素质的职业农民队伍。构建现代农业产业体系。健全安全监管检测体系，在规模生产基地试点农产品全程追溯管理，让市民吃上放心粮油菜；健全市场销售体系，鼓励发展农产品连锁配送、网上销售等新型营销方式；健全为农服务体系，重点在信息、科技、质量安全、维权护农等方面提供更完善的社会化服务。深化农村领域改革。加快农村土地确权颁证；推进农村产权交易体系建设，实现农村产权交易的常态化、公开化、规范化；引导规范土地经营权有序流转，探索土地入股、土地托管等适度规模经营方式。

提升现代服务业发展水平。聚力发展新型产业，促进服务业发展提速、比重提高、结构提升。做强电子商务。以创建国家电子商务示范城市为目标，按照电子商务“一区多点”总体框架，重点推进中国宿迁电子商务产业园“四个中心”建设；完善“政府+知名网络平台+市场主体”的电子商务发展模式，支持企业“触电上网”，推动宿迁本地产品全网销售；与京东集团等知名电商开展定制合作，发展壮大定制经济，培育新的经济增长点；精心筹办电商大会。做精文化旅游。制定旅游业发展六年行动计划，加快项王故里三期、三台山风景名胜区等旅游重点项目建设，推动骆马湖旅游度假区、洪泽湖湿地公园等景区创建升级；充分挖掘历史文化游、自然风光游等特色旅游资源，推进乡村旅游开发，打造具有宿迁地域文化特色的旅游品牌。做大健康养老。依托三台山森林公园建设康复疗养基地，发展康复保健、运动休闲等行业，创建国家级健康养老产业基地。做优现代物流。加快运河宿迁港二类口岸建设，规划建设物流园区和物流信息平台，进一步降低企业物流成本。做活现代金融。积极引进民生银行、兴业银行、招商银行来宿设立分支机构，进一步健全金融服务体系；加快推进企业上市，提高上市公司再融资能力，引导企业运用多种形式的直接融资工具，力争全年新增融资中直接融资占比提升到40%；推动融资租赁业务集聚发展，搭建各类要素交易平台，提升金融市场开放度，激发更多金融活力。

增强科技创新引领发展能力。以创建国家创新型试点城市为总领，进一步提升产业和企业自主创新能力，加快创新驱动发展步伐。强化载体平台建设。统筹推进省市级创新平台建设，打造公共技术服务平台，年内新建产业技术研究院2家以上，新增省级以上企业研发机构30家。强化产学研合作。鼓励境内外高校院所在宿设立技术转移机构和成果产业化基地，办好第十二届长三角科技论坛暨第三届江苏•宿迁生态科技博览会与产学研合作洽谈会，推进科技创新资源向宿迁集聚、成果在宿迁转化。强化产品质量提升。积极争创全国质量强市示范城市，深入开展技术标准提升工程和名牌培育集聚工程，年内新创省级以上名牌产品12个。强化科技人才建设。实施“人才引培四大工程”，年内引进国家“千人计划”和入选省“双创人才”各10名以上。

二、以文明创建为统领，增添城镇建设管理新动能

围绕创建全国文明城市，以实施“多规融合”为契机，按照规划讲统筹、建设出精品、管理重标准的要求，组织实施一批重大项目和精品工程，加快推进以人为核心的新型城镇化。

打造个性城市。全面推进总投资586亿元的中心城市139项重大项目和重点基础设施项目建设，让城市更新、更净、更美，一步步把“最美的生态、永远的时尚”化为现实。提升六类设施，打造功能配套完善的中心城市。交通设施上，继续推进道路衔接贯通，完善路网结构，加快环城北路、青海湖路、三台山大道等项目建设，同时依托高铁宿迁站规划建设高铁综合枢纽，坚持公交优先，研究推进快速公交系统，再投放1000辆公共自行车，构建更加顺畅、更加完善的公共交通服务体系；教育设施上，启动“八校”建设，优化教育布局，让更多孩子就近入学；医疗卫生设施上，支持市人民医院创三甲，市第一人民医院下半年投入运营；环卫设施上，新（改）建公厕45座，新建压缩式垃圾中转站1座、建筑垃圾转运站3座；供排水设施上，加快推进城东自来水厂、核心区雨污水管网以及城区3座污水处理厂建设，实现核心区雨污分流，保障城市供水安全；能源设施上，加快推进燃气设施、分布式能源点及CNG加气站、LNG应急气源储配站等项目建设，保障城市能源供应。加快会展中心建设，确保上半年建成投用。建设“四城一山”，打造个性特色鲜明的中心城市。围绕建设“林荫城市、彩色城市、花园城市、海绵城市”，实施104项园林绿化项目，打造一批彩色道路、花墙花廊花瀑，改造一批小游园、小广场，建设一批城市“生态水泡”、下沉式绿地、雨水湿地，让城市环境更美丽、生活更宜居；加快推进三台山森林公园扩面提质工程，精雕细琢衲田、镜湖、相思湖等景区，将衲田村、晒米台打造成为城市建设新亮点，确保以衲田为代表的一期项目“五一”开门迎宾。繁荣核心商圈，打造人气商气集聚的中心城市。在繁荣已有商业项目的同时，加快雨润广场、宿迁1897等项目建设，高品位规划建设新盛街宿迁记忆广场，年内中豪国际广场、水韵城等项目建成运营，更好地集聚和服务群众。完善市民公约，打造文明规范有序的中心城市。丰富“宿迁规矩”内涵，提升“五城同创”成果，年内创成中国人居环境奖城市。加大对店外经营、流动摊贩、三轮车营运、噪音扰民等整治力度，年内完成213个住宅小区物业整治；大力弘扬社会主义核心价值观，铸强社会文明“主心骨”；在全社会倡导“善待家人、善待他人、善待客人”的良好风尚，加快形成具有宿迁特色的文明习惯。

建设美丽县城。实施市县联动、互补融合、统筹推进，加快建设各具特色、各有亮点的美丽县城。沭阳县按照区域次中心的定位，争创国家卫生县城，打造宜居宜业宜游的美丽城市。泗阳县发挥铁路、运河等交通优势，争创国家园林县城和国家生态县，打造生态精致宜居的苏北最美县城。泗洪县做足做活大湖湿地文章，启动实施国家文明城市、国家卫生县城、国家园林县城创建，打造苏皖边界最美滨湖水韵之城。

培育特色乡村。坚持尊重历史、顺应自然，因地制宜推进乡村建设，让农村居民看得见山水、记得住乡愁、享受到优质便捷的生活服务。推进乡镇特色化发展。围绕一乡一色、一镇一品，挖掘文化内涵，培育特色产业，完善基础设施配套，提升公共服务水平，突出抓好重点中心镇建设，培育一批功能完善、特色彰显、环境优美、宜居宜业的产业重镇、风情小镇、旅游名镇。推进村庄特色化建设。加快村庄布局优化，力争一季度完成规划编制报批备案；立足“民富、村美、风气正”，梳理挑选一批具有一定历史人文、自然生态、产业特色的村庄，科学制定村庄规划，大力推进环境整治、改厕等工作，打造一批环境优美宜居、村容景致独特、乡风文明和谐的魅力田园乡村；选择有基础、有条件的村庄，再启动一批乡里中心和公租农庄建设。

推进交通基础设施建设。牢固树立“四海”意识，加快完善现代综合交通运输体系，不断提升宿迁区域联通、对外开放的水平。突破重大交通工程项目。统筹做好徐宿淮盐高铁项目开工准备工作，为上半年开工创造良好条件；加快通用机场项目前期准备工作，同步推进跑道、气象观测站等建设；继续推进徐宿连航道项目前期和争取工作；宿豫陆运河项目船闸和一期航道工程年内开工建设。加快新一轮高等级公路建设。重点推进宿扬高速宿迁段、省道330宿迁段、省道326沭阳段、省道204双沟至靳桥段续建工程，启动省道329沭阳段、省道267泗阳段、省道347等建设。加强项目包装策划。加大对上沟通协调力度，开展宿迁至南京城际铁路规划研究，争取连宿蚌铁路、新长铁路新沂至淮安段复线电气化改造等项目编入《国家“十三五”铁路发展规划》。

三、以问题和需求为导向，增添民生保障改善新动能

从群众最期盼、最急需的事情做起，下大力气解决群众关心的热点难点问题，推进总投资225亿元的30个民生实事项目，让群众分享到更多改革发展的成果。

扎实做好基础性民生。满足基本需求。加快推进区域供水和农村饮水安全工程，探索创新城乡供水管理体制和运营机制，年内全市区域供水覆盖率达85%以上；实施农村公路提档升级工程，接通“断头路”，解决好“最后一公里”问题。拓展基本服务。推进城乡公交一体化工程，年内城市公交分担率、镇村公交开通率分别达23%和80%；推进农村有线电视数字化整转，年内新增有线电视用户14.9万户，入户率提高到95%以上。

扎实做好增长性民生。在提高就业中安民。实施积极就业政策，推动稳定就业和更高质量就业，让更多群众提高工资性收入。在促进创业中富民。鼓励围绕工业配套，实施精准化创业；加快网络创业园区、孵化基地建设，年内实现网络创业载体市县乡全覆盖；办活办好“金种子”网络创业大赛，打造具有全国影响力的品牌活动。在加强保障中利民。落实城乡低保自然增长机制，推进社保扩面“幸福行动”，年内新增企业职工“五险”扩面28万人次，三大保险覆盖率达95%以上；加大棚户区改造力度，优化拆迁安置和征地补偿方式，推动拆迁实物安置为主向货币化安置为主转变、征地货币化补偿向全面保障转变，保障性安居工程从“补砖头”转向“补人头”。

扎实做好普惠性民生。促进教育均衡发展。实施学前教育五年行动计划，探索支持学前教育发展机制；实施全面改善义务教育薄弱学校办学条件工程，优化农村学校教师年龄、专业和学历结构，缩小城乡间、校际间办学差距；完善现代职业教育体系，推动职业教育特色化发展、品牌化打造；制定深化民办教育改革与发展意见，支持民办教育持续健康发展。提升医疗卫生水平。围绕构建现代医疗卫生体系，通过购买服务、契约管理，进一步优化基层公共卫生服务体系，有效落实11大类43项基本公共卫生服务项目；加快市公共卫生服务中心建设；全市1309个村全部建成标准化卫生室，新建50个社区卫生服务站，在乡镇医院试点实施、在社区全面实施基本药物制度，更好地满足群众就近就医和基本用药需求；制定出台支持民营医院发展政策，促进民营医院更好更快发展。推进文体事业繁荣发展。建设一批重点文化产业项目和特色文化创意产业园区，推进街道文化“三个一”和社区文化“五个一”建设；举办宿迁书画家晋京展、全国书法兰亭奖名家邀请展等，促进书画艺术对外交流；加快引培高层次艺术人才，集聚一批名师名家；启动三台山文创基地建设，打造高端文化艺术交流平台；实施“全民阅读”工程，打造“书香宿迁”；创新一批新民俗，让传统文化“活起来”。精心组织骆马湖国际生态四项赛，办好第四届市运会。构建养老服务体系。推进城市社区居家养老服务中心提档升级，扩大农村社区居家养老服务站覆盖面，建成运营“12349”居家养老服务平台，支持社会力量兴办或参与养老机构建设，加快构建多元化养老服务体系，年内城市社区居家养老服务实现全覆盖，50%的农村社区开展居家养老服务。

扎实做好特殊性民生。深度推进扶贫开发。强化精准式扶贫，抓好项目实施，确保完成年度扶贫开发任务；加大三大集中连片地区、黄河故道地区和淮沭河沿线地区开发推进力度，落实整体帮扶规划和扶贫菜单，年内泗洪西南岗、成子湖周边和黄墩湖滞洪区整体脱贫。兜底做好社会救助。全面提高各类特困群体的基本生活保障标准，继续提高城乡医疗救助比例，织密织好社会保障安全网；落实好残疾人社会保障政策，对重度无劳动能力残疾人实施兜底保障；扎实开展“救急难”工作，建立社会救助“一门受理、协同办理”机制，确保困难群众求助有门、受助及时。

四、以四大工程为抓手，增添生态文明建设新动能

以最高标准、最大力度和最严格的考核推进生态文明建设，重点抓好总投资146亿元的20个生态文明工程项目，促进经济发展和生态文明建设相辅相成、相得益彰。

围绕天蓝气爽深呼吸的目标，推进大气质量提升工程。抓扬尘。全面加强工地监管、道路保洁和渣土车辆管理，加大扬尘排污费征收力度，倒逼排放单位减少扬尘排放。限尾气。对农用车、过境货车及无环保绿色标志车辆实行市区全面限行，加快淘汰黄标车和老旧车辆，拆解改造一批老旧船舶。治废气。加快实施企业脱硫脱硝和除尘设施提标改造、燃煤锅炉改造，积极推进重点污染企业搬迁。用秸秆。强化全面禁烧，拓宽秸秆综合利用渠道，推进全量还田，年内综合利用率达90%。

围绕河清湖秀能游泳的目标，推进水体整治工程。治理水环境。推进污染物协同控制，着力解决城乡河道黑臭等污染问题，年内确保马陵河整治取得实质性成效。保护水源地。强化水源地建设和风险防范，完善水源地保护机制，实施取水口封闭式保护工程，提升饮用水安全保障水平。修复水生态。通过生态补水、截污、去圩坝、生态修复等方式，年内完成西民便河、古黄河等重点河流生态修复工程；加快编制骆马湖、洪泽湖保护与生态修复规划；实施中运河综合整治，提升运河沿线生态环境。

围绕平原林海好生态的目标，推进植绿护绿工程。美化城市生态。以创建国家生态园林城市为载体，加快城市河岸、湖岸和主干道两侧绿化带建设，对闲置土地和待开发裸露地实施过渡性绿化，千方百计增加城市绿量。改善乡村生态。深入开展绿化造林和全民义务植树活动，继续实施村庄绿化、绿色通道建设等工程；进一步丰富林相，更新优化树种，逐步解决杨花“七年之痒”。构建区域生态。推进五大生态走廊建设，通过实施绿化提档升级、生态修复、景观改造等工程，打造区域性大型绿色生态屏障。

围绕清洁降耗少排放的目标，推进节能减排工程。实施工程、结构和管理减排，推进城市、乡镇、农民集中居住点污水处理厂（设施）和管网建设，加强畜禽规模化养殖场和养殖小区污染防治；推广应用节能减排新技术、新产品、新装备，有序淘汰落后产能，发展绿色循环低碳经济，确保完成年度节能减排任务。

五、以减权去利为核心，增添市场主体发展新动能

按照市场化、便利化、法治化的原则，进一步深化经济体制改革，以改革的思维和手段破解瓶颈、激发活力、推动发展、造福百姓。

推进行政管理领域改革。深化商事登记制度改革。加快推进“三证合一”改革试点，上半年推开至所有县区和基层市场监管分局（所）；积极探索“一企一码”改革、个人网店全程电子化登记，创新市场主体简易注销程序，最大程度提高市场准入便利化水平；充分发挥证照联动监管平台基础支撑作用，全面构建纵横一体、完整紧密的审批监管链条。深化资格资质去行政化改革。加强与有影响力的培训机构合作，年内引进承接“两资”项目的行业协会组织不少于30家；引导培训机构、行业协会等社会组织主动对接“两资”培训项目，全年开展相关社会化培训不少于11万人次。深化行政审批制度改革。探索开展集中综合审批、集中分类评估评审试点改革，全面取消非行政许可审批事项，尽快出台权力责任清单，坚决打破“玻璃门”、“弹簧门”，彻底拆除“旋转门”；以实施注册建筑师制度为突破口，推进施工图审查市场化，深化建设领域体制改革。积极稳妥做好公车改革和不动产统一登记工作。

推进放活市场领域改革。实施政府购买服务改革。研究制定“负面清单”，除应当由政府直接提供的服务事项外，其他逐步通过购买服务实现，全年实行政府购买服务比例达到公共服务项目资金的30%左右。实施非国有资源交易改革。探索非国有资金投资项目招标人自行选择招标方式，或申请办理直接发包手续，切实提升交易效率。实施公共资源交易管理改革。全面推行行业主管部门信用考核结果、第三方信用产品在招标采购中应用，优化评标办法，力争在招标采购信用管理上实现新突破；将排污权有偿使用和交易试点扩大到所有行业，强化环境资源有价理念。

推进政府投融资领域改革。理顺管理体制。对土地收入、存量资产等有形资源，特许经营权、公共产品定价权等无形资源，公益性、非公益性和介于二者之间的第三类资产，全面进行整合，统一管理使用。转变投资方向。分类制定五大平台公司战略规划，逐步退出竞争市场和担保业务，重点投向民生、重大基础设施等领域，实现平台公司转型升级。转换融资机制。推动融资模式从市财政主导、“借用还”分离、统筹统支向企业主导、“借用还”一体化转型；探索推进市本级国有平台资产资源整合重组，实现资产管理和融资的集约化、企业化、专业化。

推进其他领域改革。全面落实好中央和省委部署的各项改革，按照市委、市政府的统一部署，扎实推进七大类30项改革，力争在资源要素配置、财政体制机制、产权制度等重点领域和关键环节上实现新突破。尊重基层首创精神，鼓励支持各地、各部门结合实际进行改革实践探索，实现改革红利最大化、持久化。

六、以民主法治为基石，增添政府依法行政新动能

坚持将依法行政作为政府履职的基本准绳，加快构建决策科学、执行坚决、监督有力的权力运行体系，推动法治政府建设再上新台阶。

增强法治意识。按照“谁主管、谁普法，谁执法、谁普法”的原则，建立机关普法责任制，切实加强法治宣传教育；落实各项学法制度，强化各级干部法律知识和法治意识；完善行政机关负责人出庭应诉制度，提高行政机关负责人出庭应诉率，倒逼各级领导干部敬畏法律、遵从法律。

推进依法决策。严格执行重大行政决策程序规定，对涉及公众重大利益的决策，引入无利害关系、无利益关联的第三方机构拟订草案；健全重大决策合法性、风险性和廉洁性审查评估机制，未经审查评估或审查评估未通过的，不提交集体讨论决定；建立决策事项实施情况评估制度，并根据评估结果进行动态调整；探索建立重大行政决策网上公开运行系统，规范决策过程和决策事项实施，提高行政效能、提升督查效果、增强决策透明度。

实施规范执法。制定针对一般性程序的行政执法程序指引和分条线的执法程序细则，构建“一总多合”的规范性程序体系。落实行政执法责任制，推进规范文明执法；支持法院、检察院依法行使司法权；完善行政处罚自由裁量基准，对行政裁量权进行规范性约束，减少自由裁量的主观操控，破除权力寻租的空间；探索综合执法改革，构建专业、高效、精简的行政执法体系。

强化法治监督。自觉接受市人大及其常委会法律监督和市政协民主监督，认真办理人大代表建议和政协委员提案；加强对下级行政机关和部门的层级监督，综合运用审计监督、行政监察、行政复议等有效手段，形成严密有效的内部监督机制；加大政务公开和政府信息公开力度，让权力在阳光下运行。

七、以预算管理为支撑，增添财政改革发展新动能

按照新《预算法》及中央关于深化预算管理制度改革的相关要求，强化预算编制的科学性、执行的约束性和管理的规范性，加快构建科学、规范、透明的财政管理体系。

深化预算改革。完善预算体系。将政府全部收支纳入预算管理，完整编制一般公共预算、政府性基金预算、国有资本经营预算和社会保险基金预算“四本账”；推进中期财政规划管理，在义务教育、卫生、社保就业、环保等重点领域开展三年滚动预算试点。推进透明预算。政府“四本账”以及部门预决算、“三公”经费预决算全口径对社会公开，主动接受社会监督；逐步公开政策制定、项目评审、资金分配、绩效评价、财政监督等决策过程和依据。硬化预算约束。按照“先有预算、后有支出，没有预算不支出”的原则，维护预算严肃性和约束力，除预算确定的支出事项外，原则上不再追加预算；加强预算执行动态监控和支出审核管理，强化重点支出项目跟踪管理，严格按制度规定支付资金。优化支出结构。落实厉行节约各项规定，完善各类经费管理制度，严控政府性楼堂馆所、财政供养人员及“三公”经费等一般性支出；保障各类重点支出，新增财力主要用于民生社会事业发展，着力解决制度碎片化问题，增强民生政策的公平性和可持续性。

规范债务管理。厘清政府债务与企业债务，将政府债务纳入预算管理，企业债务自行化解，做到谁借谁还、风险自担；健全政府债务扎口管理机制，公益性项目融资须经债务风险评定和规范性审核，坚决制止非法集资、违规担保和以BT方式举借政府债务；严格在规模限额内举债，合理控制债务余额的规模和增长速度，健全债务风险预警机制，推动融资平台公司符合条件的存量项目转化为PPP模式，减轻政府资金压力；完善权责发生制政府综合财务报告制度，完整反映政府资产负债及运行情况；加强考核问责，把政府债务作为硬指标纳入政绩考核，对脱离实际过度举债、违法违规举债或担保、违规使用债务资金、恶意逃废债务等行为，依法追究相关责任。

全面提高新常态下政府工作水平

各位代表，当前经济发展已步入新常态，经济形势发生了深层次的嬗变。为此，我们将切实把思想和认识统一到中央对形势的正确判断上来，统一到省委、省政府的最新要求上来，统一到市委、市政府的具体部署上来，坚持好的传统、好的经验、好的做法，走出思维惯性、行为惯性的困境，与时俱进转变体制机制、方式方法，真正做到观念上适应、认识上到位、方法上对路、工作上得力，牢牢把握各项工作的主动权，努力克服越来越多“既不又不、既要又要”的挑战，取得一个又一个“既能又能”的佳绩。

以思维方式的转变抢抓新机遇、实现新发展。在发展模式上树立集约思维。加快改变简单粗放的发展模式，更多地依靠科学技术进步、生产工艺改进、劳动力素质提高、管理能力提升来实现经济的可持续发展，使生产发展与资源、环境相协调。在发展速度上树立底线思维。科学确定经济增长容忍度，摆脱传统的“速度情结”，既不好高骛远，追求不切实际的增长；又时刻警惕轻视增长的危险，牢牢把握宿迁阶段性特征，推动经济增长在合理区间内取上限，始终保持高于全省、快于周边的发展态势。在发展目的上树立为民思维。不搞政绩工程和形象工程，从最小的事、最具体的事、最实在的事和最需要的地方做起，一件事情接着一件事情办，一年接着一年干，努力为群众提供更好的教育、优质的医疗、优美的环境、舒适的居住、稳定的工作、满意的收入、可靠的社会保障，让群众在改革发展中更多更好地得益受惠。

以市场作用的发挥创造新格局、激发新活力。明晰政府与市场行为边界，促进市场主体在自由竞争下自我调节和发展。在产业培育上突出市场导向。清理各类违反规定的优惠政策，设立产业发展基金，定向扶持特定产业及产业重点发展方向，对具体企业则按市场规律优胜劣汰；优化科技扶持政策，集聚科技创新资源，促进科技成果转化；探索采用风险补偿方式撬动金融资本加大对产业发展支持力度。在项目建设上吸收市场资源。深化投资领域审批制度改革，减少投资办理环节，拓宽社会投资领域，创新投资合作方式，通过市场换投资、设备换投资等模式，吸引更多项目落户宿迁；在交通物流、园区建设、污废处理、社会事业等重点领域推广PPP模式，构建合理的收益共享、风险共担机制，把政府的政策导向和社会资本的运营效率结合起来，吸引更多社会资本参与宿迁建设发展。在要素配置上发挥市场作用。深化水气热等资源要素价格改革，年内实现全市水气热价格市场主导；继续开展大用户直购电试点；探索建立土地先租后让弹性出让机制、企业用地亩均效益评价机制和开发区建设导则，推进节约集约用地“双提升”。

以治理模式的完善适应新情况、凝聚新共识。更加注重社会自身调节和自我管理，让各种社会力量共同发挥作用。加强社会组织培育。从制度层面解决行业协会、商会法律地位和权责关系，理顺管理机制，促进职能重构；以购买服务等方式，培育发展一批具备承接政府职能转移的行业协会、商会和中介机构，更好地助推产业发展。健全信用管理体系。扩大信用产品应用范围，年内市域范围实现在重点部门和重点领域全面推行第三方信用报告制度，加快构建“守信激励、失信惩戒”的管理机制。全面推行“政社联建”。创新基层社会管理，推行“一家双联三议事”民主管理制度，实现政府治理与基层群众自治的有效衔接和协同联建。

以政府职能的强化落实新举措、满足新需求。认真履行政府职能，把该做的事做实、做好、做出成效。优化公共服务。加强公用基础设施、教育卫生设施、文化体育设施建设，形成完善的公共服务体系。严肃行政执法。围绕安全生产、环境保护、劳动保障、城市管理等行政执法重点领域，加大执法检查力度，坚持严厉、公平、公正执法，做到见违必纠、纠违必罚。维护市场秩序。健全完善监管体系和责任追究制度，推动执法力量重心下移；加强市场监督管理，持续开展假冒伪劣、食品药品安全等专项整治行动，营造公平竞争、健康有序的市场环境。强化安全生产。坚持“党政同责、一岗双责、齐抓共管”，严格落实安全生产监督管理措施，严防重特大安全生产事故发生。促进社会和谐。坚持系统治理、依法治理、综合治理、源头治理，构建立体化、现代化的社会治安防控体系；深化“平安宿迁”、“法治宿迁”建设，优化社会治安环境，不断提升群众安全感；加强信访稳定工作，完善公共法律服务体系，健全政府法律顾问和公职律师制度，进一步提高合理合法解决群众诉求的能力和水平。

加强和改进政府工作作风

各位代表，新的形势和任务对政府工作提出了更高要求。我们将巩固提升群众路线教育实践活动成果，加快转变政府职能，改进工作作风，提高行政效能，不断增强政府执行力和公信力。

注重学习调研。适应新形势、新使命、新要求，从习总书记重要讲话中获取强大思想武器，从基层和群众中汲取智慧和营养，从实践中找到破解难题的钥匙，使政府各项工作体现时代性、遵循规律性、富有创造性。大力倡导学习调研之风，创设“星期三论坛”，完善政府领导集体学习制度、专家授课制度，健全公务员任前和任中培训等制度，加强对新理论、新政策、新知识的研究，全面提升政府工作人员的情商和“警商”，增强业务素质和能力，成为引领新常态下各项工作的行家里手。

坚持勤政为民。时刻牢记肩负的历史重任，始终保持忘我的工作激情，主动担当，积极作为，以抓落实的“快”来保证发展速度的“快”，以抓落实的“好”来保证发展质量的“好”。组织开展专项整治活动，严厉查处有令不行、有禁不止的行为，严厉查处在贯彻中央、省、市要求和政策上搞选择性执行、象征性落实的行为，严厉查处对群众、企业和基层组织的正当诉求不作为、慢作为的行为；继续做好“四风”突出问题整改工作，逐一解决“群众不满意”事项；加强行政效能监察，以更严格的目标管理和监察问责，推动政务服务提速、提质、提效。

永葆清正廉洁。深入贯彻中央八项规定、省委十项规定和市委十条禁令，严格落实党风廉政建设责任制，不断强化惩治和预防腐败体系建设。构建“不想腐”的自律机制，以领导干部为重点，以理想教育为核心，加强道德信念、廉政警示、法规法纪教育，让广大干部心有所畏、言有所戒、行有所止，把廉洁从政作为自觉追求。完善“不能腐”的制约机制，坚持用制度管权管事管人，把权力关进制度“笼子”，从源头上预防腐败；推动公共资金、国有资产资源和领导干部履行经济责任情况审计全覆盖，进一步发挥好审计监督作用。强化“不敢腐”的惩戒机制，始终保持严惩腐败高压态势，让顶风违纪者得不偿失，让心存侥幸者望而生畏，加快形成干部清正、政府清廉、政治清明的良好政务环境。

各位代表！我们深知，全市人民对宿迁发展充满着企盼和期待，对美好明天有着更多的憧憬和向往；我们也深知，千里之远需跬步以至，发展大业在日积月累。建设经济强、百姓富、环境美、社会文明程度高的新宿迁，需要我们从改革发展的一点一滴做起，从群众期盼的一情一事做起，从每一个人的一言一行做起。让我们在市委的坚强领导下，在市人大、市政协和社会各界的监督支持下，同心同德，开拓创新，务实奋进，为推动宿迁经济社会转型发展、绿色发展、创新发展，谱写全面小康社会建设新篇章而努力奋斗！
